# *Passiflora edulis*: An Insight Into Current Researches on Phytochemistry and Pharmacology

**DOI:** 10.3389/fphar.2020.00617

**Published:** 2020-05-20

**Authors:** Xirui He, Fei Luan, Yan Yang, Ze Wang, Zefeng Zhao, Jiacheng Fang, Min Wang, Manhua Zuo, Yongsheng Li

**Affiliations:** ^1^Department of Bioengineering, Zhuhai Campus of Zunyi Medical University, Zhuhai, China; ^2^Department of Pharmacology, College of Pharmacy, Chengdu University of Traditional Chinese Medicine, Chengdu, China; ^3^College of Life Sciences, Northwest University, Xi’an, China; ^4^Department of Nursing, Zhuhai Campus of Zunyi Medical University, Zhuhai, China; ^5^Department of Pharmacy, Honghui Hospital, Xi’an Jiaotong University, Xi’an, China

**Keywords:** *Passiflora edulis*, passion fruit, polyphenols, nutritional components, antioxidant activities

## Abstract

*Passiflora edulis*, also known as passion fruit, is widely distributed in tropical and subtropical areas of the world and becomes popular because of balanced nutrition and health benefits. Currently, more than 110 phytochemical constituents have been found and identified from the different plant parts of *P. edulis* in which flavonoids and triterpenoids held the biggest share. Various extracts, fruit juice and isolated compounds showed a wide range of health effects and biological activities such as antioxidant, anti-hypertensive, anti-tumor, antidiabetic, hypolipidemic activities, and so forth. Daily consumption of passion fruit at common doses is non-toxic and safe. *P. edulis* has great potential development and the vast future application for this economically important crop worldwide, and it is in great demand as a fresh product or a formula for food, health care products or medicines. This mini-review aims to provide systematically reorganized information on physiochemical features, nutritional benefits, biological activities, toxicity, and potential applications of leaves, stems, fruits, and peels of *P. edulis*.

## Introduction

**Figure 1:**
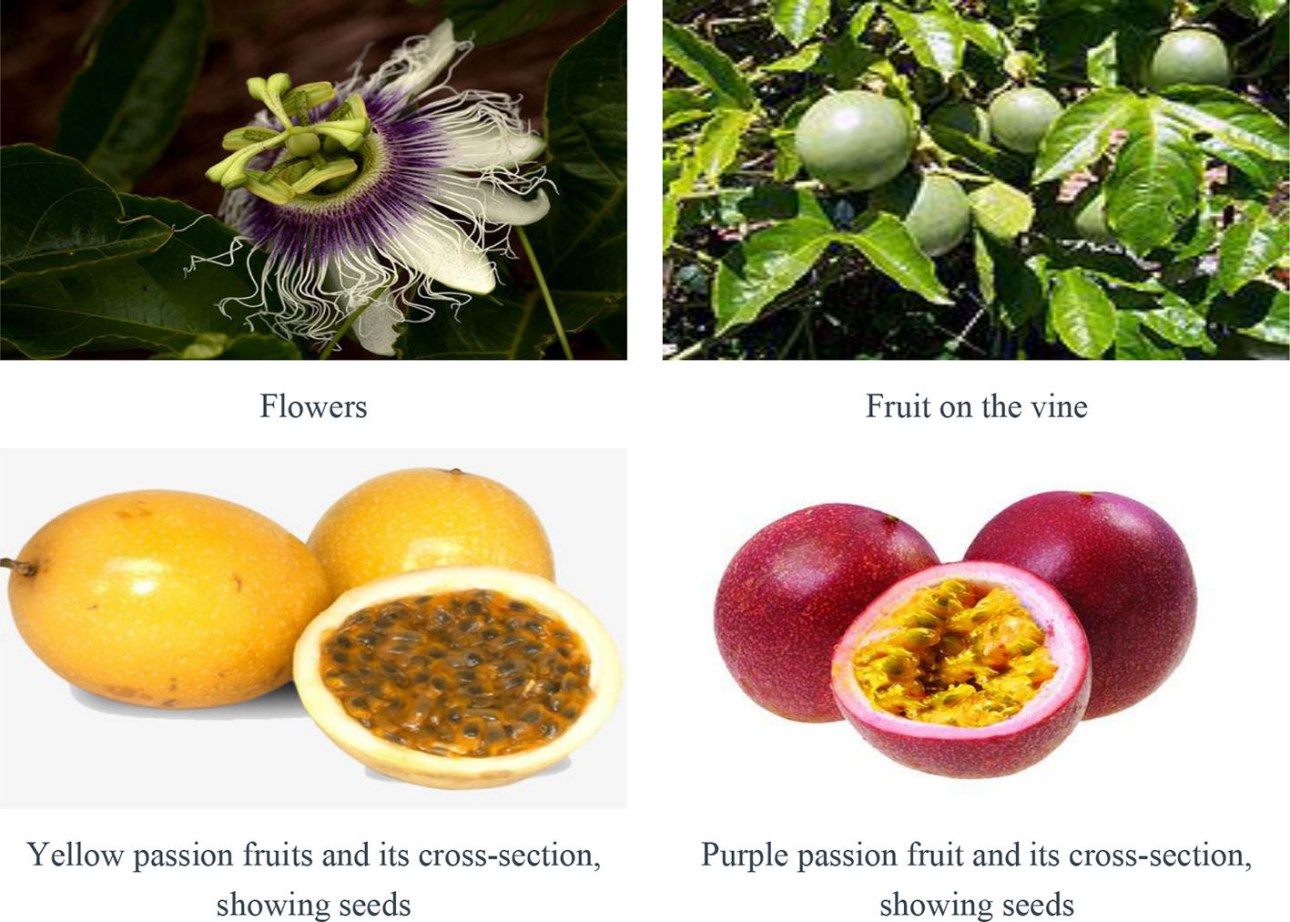
Flowers, leaves, and fruits of *P. edulis* (https://image.baidu.com/).

The genus *Passiflora*, comprising about 500 species, is the largest in family Passifloraceae. Among which, the *Passiflora edulis* are stands out because of its economic and medicinal importance. (Dhawan et al., 2004). It is widely planted in tropical and subtropical regions in several parts of the world, especially in South America, Caribbean, south Florida, South Africa, and Asia (Zhang et al., 2013; Yuan et al., 2017; Hu et al., 2018). There are seven varieties provided in The Plant List including *P. edulis* Sims, *P. edulis* f. *edulis*, *P. edulis* f. *flavicarpa* O. Deg., *P. edulis* var. *kerii* (Spreng.) Mast., *P. edulis* var. *pomifera* (M. Roem.) Mast., *P. edulis* var. *pomifera* (M. Roem.) Mast., *P. edulis* var. *rubricaulis* (Jacq.) Mast., and *P. edulis* var. *verrucifera* (Lindl.) Mast (The Plant List, 2013). Among them, the yellow-fruited *P. edulis* f. *flavicarpa* O. Deg. and the purple-fruited type, *P. edulis* Sims are the two main and common varieties with considerable economic importance (Zucolotto et al., 2009; Cazarin et al., 2016). The yellow passion fruit is 6–12 cm long and 4–7 cm in diameter. The peel is bright yellow, hard, and thick. The seeds are brown. The pulp is acidic and has a strong aromatic flavor. The purple passion fruit is relatively small in size (4–9 cm long and 3.5–7 cm in diameter). Its peel is purple and seed is black (Narain et al., 2010). Their relevant pictures are listed in **Figure 1**.

In recent years, with the considerable work done on *P. edulis* development, there has been an increasing interest to utilize passion fruit for human consumption due to the eating quality of its fruits, juiciness, attractive nutritional values, essential health benefits, and the people’s choice (Cazarin et al., 2016; Lima et al., 2016; Pereira et al., 2019). Passion fruit, also well known as “the king of fruits”, “maracujá”, “love fruit”, and “fruitlover”, is frequently eaten freshly or squeezed for juice. Meanwhile, a range of products made with passion fruit has been developed including cake, ice cream, jam, jelly, yoghurt, compound beverage, tea, wine, vinegar, soup-stock, condiment sauce, and so on. Passion fruit is also used as traditional folk medicines and cosmetic moisturizing agent in many countries (Xu et al., 2016). In China, the purple passion fruit has been adapted for the cultivation in the warm climate of Jiangsu, Fujian, Taiwan, Hunan, Guangdong, Hainan, Guangxi, Guizhou, Yunnan, and so forth. The purple passion fruit consumption occurs mainly in the form of fresh fruit and fruit juice. According to *ZhongHuaBenCao* (Simplified Chinese: 中华本草) records, it is sweet, sour in flavour, and highly aromatic, and acts on the heart and large intestine meridians. *ZhongHuaBenCao* recommends its dosage between 10 and 15 g when taken orally as decoct soup for treatment of cough, hoarseness, constipation, dysmenorrhea, arthralgia, dysentery, insomnia, and so forth. In Brazil, the yellow passion fruit is most commonly used for the preparation of soft drinks and as a remedy in folk medicine, like juices nectars, tinctures or tablets. Today, other parts of *P. edulis* have also been developed and utilized in many countries. The leaves of *P. edulis* with highly appreciated and pleasant taste are widely used as sedatives or tranquilizers in the United States and European countries. The flowers are large and beautiful, and can be used as garden ornamental plants. The peels, characterized by high levels of polyphenols, fibers and trace elements, have been widely used for making wine or tea, cooking dishes, extracting pectin and medicinal ingredients, and processing feed. The seeds are edible, and high in protein and oil (mainly composed of linoleic acid, oleic acid, and palmitic acid).

Apart from being a food item, a variety of pharmaceutical products based on ingredients have also be developed and used in folk medicine. The principal components of *P. edulis* include polyphenols, triterpenes, and its glycosides, carotenoids, cyanogenic glycosides, polysaccharides, amino acids, essential oils, microelements, and so forth (Xu et al., 2013; Zhang et al., 2013; Yuan et al., 2017; Hu et al., 2018). Among these compounds, the most reported are luteolin, apigenin, and quercetin derivatives. Most importantly, passion fruit contains nutritionally valuable compounds like vitamin C, dietary fiber, B vitamins, niacin, iron, phosphorus, and so forth. A wide range of *in vitro* and *in vivo* pharmacological studies have revealed various promising bioactivities of *P. edulis*, such as antioxidant, antimicrobial, anti-inflammatory, anti-hypertensive, hepatoprotective and lung-protective activities, anti-diabetic, sedative, antidepressant activity, and anxiolytic-like actions (Nayak and Panda, 2012; Kinoshita et al., 2013; Silva et al., 2015; Dzotam et al., 2016; Zhang et al., 2016; Panelli et al., 2018). Most of these effects are consistent with those observed for *P. edulis* in traditional and folk medicine, and these pharmacological actions are thought to be mostly mediated *via* the existed bioactive components including polyphenol, triterpenes, and polysaccharides. Several researchers have reviewed the botany, chemistry, and pharmacological reports of the *Passiflora* genus (Dhawan et al., 2004; Corrêa et al., 2016). However, to date, no comprehensive review concerning the information on the chemical and biological properties of *P. edulis* is available.

In this mini-review, we intend to systematically summarize the recent advances in knowledge about chemical and biological activities of different parts of *P. edulis* (fruit, stems, leaves, and peel). The extraction methods and purification procedures for polysaccharides, processing passion fruit for formulation and production of food product is also reviewed. Future research directions on how to better utilize and develop passion fruit are suggested.

## Physicochemical and Structural Features

The major nutrient components of *P. edulis* include dietary fiber, carbohydrates, lipids, carboxylic acids, polyphenols, volatile compound, protein and amino acids, vitamins, mineral, and so forth. (**Table 1**). To date, more than 110 kinds of chemical constituents have been isolated and identified from the *P. edulis*. Among them, flavonoids, triterpenoids, and carotenoids are the primary types. Methods for determination of main chemical components from *P. edulis* are shown in **Table 2**. The main monomeric compounds are summarized and compiled in **Table 3**.

**Table 1:** Nutritional composition of purple and yellow passion fruit juice (USDA Food Composition Databases, 2019).

Nutrient	Unit	Purple passion fruit juice, raw	Yellow passion fruit juice, raw
		Value per 100 g	Value per 100 g
Proximates			
Water	g	85.62	84.21
Energy	kcal	51	60
Protein	g	0.39	0.67
Total lipid (fat)	g	0.56	0.18
Carbohydrate, by difference	g	13.6	14.45
Fiber, total dietary	g	0.2	0.2
Sugars, total	g	13.4	14.25
Minerals			
Calcium, Ca	mg	4	4
Iron, Fe	mg	0.24	0.36
Magnesium, Mg	mg	17	17
Phosphorus, P	mg	13	25
Potassium, K	mg	278	278
Sodium, Na	mg	6	6
Zinc, Zn	mg	0.05	0.06
Copper, Cu	mg	0.053	0.05
Selenium, Se	µg	0.1	0.1
Vitamins			
Vitamin C, total ascorbic acid	mg	29.8	18.2
Thiamin	mg	0.000	0.000
Riboflavin	mg	0.131	0.101
Niacin	mg	1.46	2.240
Vitamin B-6	mg	0.05	0.060
Folate, DFE	µg	7	8
Vitamin B-12	µg	0.00	0.00
Vitamin A, RAE	µg	36	47
Vitamin A, IU	IU	717	943
Vitamin E (alpha-tocopherol)	mg	0.01	0.01
Vitamin D (D2 + D3)	µg	0.0	0.0
Vitamin D	IU	0	0
Vitamin K (phylloquinone)	µg	0.4	0.4
Lipids			
Fatty acids, total saturated	g	0.004	0.015
Fatty acids, total monounsaturated	g	0.006	0.022
Fatty acids, total polyunsaturated	g	0.029	0.106

**Table 2:** Methods for determination of chemical components of *P. edulis*.

Chemical components	Plant part	Location and number of geno-type	Better methods	Major findings	Reference
Polyphenol	Pulp	Brazil, yellow passion fruit	QuEChERS method combined with C18 as d-SPE clean-up sorbent/UHPLC-MS-MS	Quercetin, rutin, 4-hydroxybenzoic, chlorogenic, ferulic, vanillic, caffeic, *trans-*cinammic, and *p*-coumaric acids, and the most abundant phenolic components were quercetin and vanillic acid.	Rotta et al., 2019
Volatile compounds	Pulp	Brazil, yellow passion fruit	Dynamic headspace/GC-MS and GC-O analysis	64 volatile compounds, and mainly include esters, alcohols, terpenes, aldehydes, and ketones, and so forth.	Janzantti et al., 2012
Carbohydrate	Pulp	France, passion fruit (unknown)	Acid hydrolysis, NaOH (2 M), Fehling solution	13.70 g glucose equivalent/100 g	Septembre-Malaterre et al., 2016
Carotenoid	Pulp	France, passion fruit (unknown)	UV-vis spectrophotometry at 450 nm	3.83 mg β-carotene equivalent/100 g	Septembre-Malaterre et al., 2016
Vitamin C	Pulp	France, passion fruit (unknown)	2,6-dichloro-phenol-indophenol titrimetric method	44.40 mg ascorbic acid equivalent/100 g	Septembre-Malaterre et al., 2016
Polyphenol	Pulp	France, passion fruit (unknown)	Folin-Ciocalteu assay	286.60 mg gallic acid equivalent/100 g	Septembre-Malaterre et al., 2016
Flavonoid	Pulp	France, passion fruit (unknown)	Colorimetric assay	70.10 mg quercetin equivalent/100 g	Septembre-Malaterre et al., 2016
Polyphenol	Fresh green leaves	Sri Lanka, passion fruit (unknown)	70% (vol/vol) acetone/alkaline hydrolysis/HPLC	The total soluble, insoluble-bound phenolic compounds and total flavonoid from the extracts were 511.20 mmol/g (gallic acid equivalent), 66.62 mmol/g (gallic acid equivalent), and 111.69 mmol/g (rutin equivalent).	Gunathilake et al., 2018a
Polyphenol	Seeds	Brazil, yellow passion fruit	70% ethanol at 80°C for 30 min	31.20 mg/g (gallic acid equivalent), and the major component was piceatannol (36.80 mg/g).	de Santana et al., 2017
Polyphenol	Peel	Brazil, yellow passion fruit	Ultrasound-assisted or Pressurized solvent extraction (ethanol at 60:40)/LC-DAD-ESI-MS	4.67 mg/g (gallic acid equivalent). and mainly include orientin, orientin-7-*O*-glucoside, vitexin and isoorientin.	de Souza et al., 2018
Polyphenol	Ripe fruits	Panama, passion fruit (unknown)	Methanol	81 mg/100 g (Gallic acid equivalent) fresh weight	Murillo et al., 2012
Oil	Seeds	Brazil, yellow passion fruit	SE using *n*-hexane as solvent	High tocopherol and fatty acid like palmitic, stearic, oleic, linoleic, α-Linolenic, behenic, caprylic, and aproic	Pereira et al., 2019
Bound terpenoids	Juice	Australia, purple passion fruit	Almond glycosidase hydrolysis with C_18_ isolates (MeOH elution), HRGC-MS	15 C_13_ norterpenoid aglycons, and the main terpenoids are 4-hydroxy-β-ionol, 4-oxo- β -ionol, 4-hydroxy-7,8-dihydro-β-ionol, 4-oxo-7,8-dihydro- β -ionol, 3-oxo-α-ionol, isomeric 3-oxoretro-α-ionols, 3-oxo-7,8-dihydro -α-ionol, 3-hydroxy-1,1, 6-trimethyl-1,2,3, 4-tetrahydronaphthalene, vomifoliol, and dehydrovomifoliol, and so forth.	Winterhalter, 1990

**Table 3:** Chemical Components of *P. edulis*.

NO.	Compounds	CAS number	Formula	Resources	Ref.
***Flavonoids***					
1	Quercetine	117-39-5	C_15_H_10_O_7_	A	Lutomski et al., 1975
2	Rutin	153-18-4	C_27_H_30_O_16_	A	Lutomski et al., 1975
3	Vitexina	3681-93-4	C_21_H_20_O_10_	A	Lutomski et al., 1975
4	Isoorientin	4261-42-1	C_21_H_20_O_11_	A	Lutomski et al., 1975
5	Saponarin	20310-89-8	C_27_H_30_O_15_	A	Lutomski et al., 1975
6	Homovitexin	38953-85-4	C_21_H_20_O_10_	A	Lutomski et al., 1975
7	Luteolin-6-C-chinovoside	132368-05-9	C_21_ H_20_ O_10_	A	Mareck et al., 1991
8	Luteolin-6-C-fucoside	138810-81-8	C_21_ H_20_ O_10_	A	Mareck et al., 1991
9	Orientin	28608-75-5	C_21_ H_20_ O_11_	A	Moraes et al., 1997
10	Myrtillin	50986-17-9	C_21_ H_21_ O_12_^+^	A	Kidoey et al., 1997
11	Petunidin 3-glucoside	71991-88-3	C_22_ H_23_ O_12_^+^	A	Kidoey et al., 1997
12	Cyanidin 3-glucoside	47705-70-4	C_21_ H_21_ O_11_^+^	A	Kidoey et al., 1997
13	Callistephin	47684-27-5	C_21_ H_21_ O_10_^+^	A	Kidoey et al., 1997
14	Cyanidin 3-(6″-malonylglucoside)	171828-62-9	C_24_ H_23_ O_14_^+^	A	Kidoey et al., 1997
15	Pelargonidin 3-(6″-malonylglucoside)	165070-68-8	C_24_ H_23_ O_13_^+^	A	Kidoey et al., 1997
16	Delphinidin 3-(6″-malonylglucoside)	478693-96-8	C_24_ H_23_ O_15_^+^	A	Kidoey et al., 1997
17	1-Benzopyrylium, 3-[[6-O-(carboxyacetyl)-β-D-glucopyranosyl]oxy]-2-(3,4-dihydroxy-5-methoxyphenyl)-5,7-dihydroxy-	687619-89-2	C_25_ H_25_ O_15_^+^	A	Kidoey et al., 1997
18	Idein	142506-26-1	C_21_ H_21_ O_11_^+^	A	Chang and Su, 1998
19	Luteolin	491-70-3	C_15_ H_10_ O_6_	B	Coleta et al., 2006
20	Lonicerin	25694-72-8	C_27_ H_30_ O_15_	B	Coleta et al., 2006
21	Vitexin, 4′-rhamnoside	32426-34-9	C_27_ H_30_ O_14_	C	Zhou et al., 2009
22	Spinosin	72063-39-9	C_28_ H_32_ O_15_	B	Zucolotto et al., 2009
23	Vicenin	23666-13-9	C_27_ H_30_ O_15_	B	Zucolotto et al., 2009
24	6,8-di-C-glycosylchrysin	850621-76-0	C_27_ H_30_ O_14_	B	Zucolotto et al., 2009
25	Chrysin 6-*C*-β-rutinoside	1488426-52-3	C_27_ H_30_ O_13_	B	Zhang et al., 2013
26	Chrysin 7-glucoside	31025-53-3	C_21_ H_20_ O_9_	D	Xu et al., 2013
27	7-[[4-*O*-(6-Deoxy-α-L-mannopyranosyl)-β-D-glucopyranosyl]oxy]-5-hydroxy-2-phenyl-4*H*-1-benzopyran-4-one	378782-33-3	C_27_ H_30_ O_13_	D	Xu et al., 2013
28	Luteolin 8-*C*-β-digitoxopyranoside	951126-36-6	C_21_ H_20_ O_9_	D	Xu et al., 2013
29	7-De-*O*-methylaciculatin	1355022-31-9	C_21_ H_20_ O_8_	D	Xu et al., 2013
30	8-*C*-β-D-Boivinopyranosylapigenin	1355022-34-2	C_21_ H_20_ O_8_	D	Xu et al., 2013
31	Luteolin 8-*C*-β-digitoxopyranosyl-4′-*O*-β-D-glucopyranoside	1402209-61-3	C_27_ H_30_ O_14_	D	Xu et al., 2013
32	Luteolin 8-*C*-β-boivinopyranoside	1402209-62-4	C_21_ H_20_ O_9_	D	Xu et al., 2013
33	Chrysin-8-*C*-(2″-*O*-6-deoxy-α-D-glucopyranosyl)-β-D-glucopyranoside	2171100-31-3	C_27_ H_30_ O_13_	E	Hu et al., 2018
***Triterpenoids***					
34	Passiflorin	1392-82-1	C_37_ H_60_ O_12_	A	Bombardelli et al., 1975
35	Cyclopassifloic acid E	301540-74-9	C_31_ H_52_ O_8_	D	Yoshikawa et al., 2000b
36	Cyclopassifloic acid F	301540-76-1	C_31_ H_52_ O_7_	D	Yoshikawa et al., 2000b
37	Cyclopassifloic acid G	301540-77-2	C_31_ H_52_ O_7_	D	Yoshikawa et al., 2000b
38	Cyclopassifloside VII	301540-80-7	C_37_ H_62_ O_13_	D	Yoshikawa et al., 2000b
39	Cyclopassifloside VIII	301540-81-8	C_37_ H_62_ O_12_	D	Yoshikawa et al., 2000b
40	Cyclopassifloside X	301540-82-9	C_37_ H_62_ O_12_	D	Yoshikawa et al., 2000b
41	Cyclopassifloside IX	301644-33-7	C_43_ H_72_ O_17_	D	Yoshikawa et al., 2000b
42	Cyclopassifloside XI	301644-34-8	C_43_ H_72_ O_17_	D	Yoshikawa et al., 2000b
43	Passifloric acid	64147-49-5	C_31_ H_50_ O_7_	D	Yoshikawa et al., 2000a
44	Cyclopassifloic acid B	292167-35-2	C_31_ H_52_ O_6_	D	Yoshikawa et al., 2000a
45	Cyclopassifloic acid C	292167-36-3	C_31_ H_52_ O_7_	D	Yoshikawa et al., 2000a
46	Cyclopassifloside IV	292167-41-0	C_37_ H_62_ O_12_	D	Yoshikawa et al., 2000a
47	Cyclopassifloside V	292167-42-1	C_43_ H_72_ O_17_	D	Yoshikawa et al., 2000a
48	Cyclopassifloside VI	292167-43-2	C_36_ H_58_ O_11_	D	Yoshikawa et al., 2000a
49	Cyclopassifloic acid D	292167-37-4	C_30_ H_48_ O_6_	D	Yoshikawa et al., 2000a
50	Cyclopassifloside II	292167-39-6	C_37_ H_62_ O_11_	D	Yoshikawa et al., 2000a
51	Cyclopassifloside I	292167-38-5	C_37_ H_62_ O_12_	D	Yoshikawa et al., 2000a
52	Cyclopassifloside III	292167-40-9	C_43_ H_72_ O_16_	D	Yoshikawa et al., 2000a
53	Cyclopassifloic acid A	292167-34-1	C_31_ H_52_ O_7_	D	Yoshikawa et al., 2000a
54	3β,16β-diacetoxyurs-12-ene	920957-33-1	C_34_ H_54_ O_4_	F	Yoshikawa et al., 2000a
55	(3β,5α,8α,22*E*)-5,8-Epidioxyergosta-6,22-dien-3-ol	2061-64-5	C_28_ H_44_ O_3_	C	Zhou et al., 2009
56	(31*R*)-31-*O*-Methylpassiflorine	1491979-55-5	C_38_ H_62_ O_12_	B	Zhang et al., 2013
57	(31S)-31-O-Methylpassiflorine	1491979-58-8	C_38_ H_62_ O_12_	B	Zhang et al., 2013
58	(31*R*)-Passiflorine	1492023-84-3	C_37_ H_60_ O_12_	B	Zhang et al., 2013
59	(31S)-Passiflorine	1492023-87-6	C_37_ H_60_ O_12_	B	Zhang et al., 2013
60	Cyclopassifloside XII	1595294-85-1	C_37_ H_60_ O_12_	D	Wang et al., 2013
61	Cyclopassifloside XIII	1595294-86-2	C_43_ H_72_ O_17_	D	Wang et al., 2013
62	β-Sitostenone	1058-61-3	C_29_ H_48_ O	B	Yuan et al., 2017
***Alkaloids***					
63	Harmidine	304-21-2	C_13_ H_14_ N_2_ O	A	Lutomski et al., 1975
64	Harmine	442-51-3	C_13_ H_12_ N_2_ O	A	Lutomski et al., 1975
65	Harmane	486-84-0	C_12_ H_10_ N_2_	A	Lutomski et al., 1975
66	Harmol	487-03-6	C_12_ H_10_ N_2_ O	A	Lutomski et al., 1975
67	*N*-*trans*-Feruloyltyramine	66648-43-9	C_18_ H_19_ N O_4_	B	Yuan et al., 2017
68	*cis*-*N*-Feruloyltyramine	80510-09-4	C_18_ H_19_ N O_4_	B	Yuan et al., 2017
***Sulforaphanes***					
69	3-(Methylthio)-1-hexanol; 3-(Methylthio)hexanol	51755-66-9	C_7_ H_16_ O S	A	Winter et al., 1976
70	*cis*-2-Methyl-4-propyl-1,3-oxathiane	59323-76-1	C_8_ H_16_ O S	A	Winter et al., 1976
71	(±)-*trans*-2-Methyl-4-propyl-1,3-oxathiane	59324-17-3	C_8_ H_16_ O S	A	Winter et al., 1976
72	Ethanethioic acid, S-[1-[2-(acetyloxy)ethyl]butyl] ester	136954-25-1	C_10_ H_18_ O_3_ S	A	Engel and Tressl, 1991
73	Butanoic acid, 3-[(1-oxobutyl)thio]hexyl ester	136954-26-2	C_14_ H_26_ O_3_ S	A	Engel and Tressl, 1991
74	Hexanoic acid, 3-[(1-oxohexyl)thio]hexyl ester	136954-27-3	C_18_ H_34_ O_3_ S	A	Engel and Tressl, 1991
***Carotenoids***					
75	Violaxanthin	126-29-4	C_40_ H_56_ O_4_	A	Mercadante et al., 1998
76	β-Cryptoxanthin	472-70-8	C_40_ H_56_ O	A	Mercadante et al., 1998
77	Neurosporene	502-64-7	C_40_ H_58_	A	Mercadante et al., 1998
78	Lycopene	502-65-8	C_40_ H_56_	A	Mercadante et al., 1998
79	Mutatochrome	515-06-0	C_30_ H_40_ O_2_	A	Mercadante et al., 1998
80	β-Citraurin	650-69-1	C_30_H_40_O_2_	A	Mercadante et al., 1998
81	Prolycopene	2361-24-2	C_40_ H_56_	A	Mercadante et al., 1998
82	β-Carotene	7235-40-7	C_40_ H_56_	A	Mercadante et al., 1998
83	Phytoene	13920-14-4	C_40_ H_64_	A	Mercadante et al., 1998
84	Neoxanthin	14660-91-4	C_40_ H_56_ O_4_	A	Mercadante et al., 1998
85	Phytofluene	27664-65-9	C_40_ H_62_	A	Mercadante et al., 1998
86	Antheraxanthin	68831-78-7	C_40_ H_56_ O_3_	A	Mercadante et al., 1998
87	ζ-Carotene	72746-33-9	C_40_ H_60_	A	Mercadante et al., 1998
***Other compounds***					
88	Prunasin	99-18-3	C_14_ H_17_ N O_6_	G	Spencer and Seigler, 1983
89	Benzyl alcohol *O*-α-L-arabinopyranosyl-(1→6)-β-D-glycopyranoside	148031-67-8	C_18_ H_26_ O_10_	A	Chassagne et al., 1996
90	3-Methyl-2-buten-1-yl 6-O-α-L-arabinopyranosyl-β-D-glucopyranoside	175737-84-5	C_16_ H_28_ O_10_	A	Chassagne et al., 1996
91	1-Ethenyl-1,5-dimethyl-4-hexen-1-yl 6-*O*-α-L-arabinopyranosyl-β-D-glucopyranoside	175892-12-3	C_21_ H_36_ O_10_	A	Chassagne et al., 1996
92	3-Oxo-α-ionol	34318-21-3	C_13_ H_20_ O_2_	A	Herderich and Winterhalter, 1991
93	Benzoic acid, 2-[[2,3,4-tri-*O*-acetyl-6-*O*-(2,3,4-tri-*O*-acetyl-6-deoxy-α-L-mannopyranosyl)-β-D-glucopyranosyl]oxy]-, methyl ester	191273-45-7	C_32_ H_40_ O_18_	A	Chassagne et al., 1997
94	Benzoic acid, 2-[[6-*O*-(6-deoxy-α-L-mannopyranosyl)-β-D-glucopyranosyl]oxy]-, methyl ester	191273-46-8	C_20_ H_28_ O_12_	A	Chassagne et al., 1997
95	Cyanogenic β-rutinoside	215583-49-6	C_20_ H_27_ N O_10_	A	Chassagne and Crouzet, 1998
96	Phenylmethyl β-D-allopyranoside	354807-69-5	C_13_ H_18_ O_6_	A	Christensen and Jaroszewski, 2001
97	Passiedulin	354814-09-8	C_14_ H_17_ N O_6_	A	Seigler et al., 2002
98	Sambunigrin	99-19-4	C_14_ H_17_ N O_6_	A	Seigler et al., 2002
99	Benzeneacetonitrile, α-(β-D-allopyranosyloxy)-, (αS)-	474075-97-3	C_14_ H_17_ N O_6_	A	Seigler et al., 2002
100	Passiflactone	1130942-01-6	C_8_ H_8_ O_4_	A	Lu et al., 2007
101	Amygdalin	29883-15-6	C_20_ H_27_ N O_11_	B	Zhang et al., 2013
102	Roseoside	54835-70-0	C_19_ H_30_ O_8_	B	Zhang et al., 2013
103	*p*-Hydroxybenzoic acid	99-96-7	C_7_ H_6_ O_3_	B	Yuan et al., 2017
104	Vanillic acid	121-34-6	C_8_ H_8_ O_4_	B	Yuan et al., 2017
105	Syringic acid	530-57-4	C_9_ H_10_ O_5_	B	Yuan et al., 2017
106	(+)-Syringaresinol	21453-69-0	C_22_ H_26_ O_8_	B	Yuan et al., 2017
107	4-Acetyl-3,5-dimethoxy-*p*-quinol	211192-56-2	C_10_ H_12_ O_5_	B	Yuan et al., 2017
108	α-Tocoquinone	7559-04-8	C_29_ H_50_ O_3_	A	Hu et al., 2018
109	Prulaurasin	138-53-4	C_14_ H_17_ N O_6_	A	Hu et al., 2018
110	Citrusin A	105279-09-2	C_26_ H_34_ O_12_	A	Hu et al., 2018
111	Citrusin B	105279-10-5	C_27_ H_36_ O_13_	A	Hu et al., 2018
112	Citrusin G	2173403-45-5	C_28_ H_38_ O_12_	A	Hu et al., 2018
113	*trans*-Coniferin	124151-33-3	C_16_ H_22_ O_8_	A	Hu et al., 2018
114	Icariside E_5_	126176-79-2	C_26_ H_34_ O_11_	A	Hu et al., 2018
115	Alangioside A	156199-49-4	C_19_ H_34_ O_8_	A	Hu et al., 2018
116	Longifloroside B	175556-09-9	C_27_ H_36_ O_13_	A	Hu et al., 2018
117	Hyuganoside IIIa	838845-07-1	C_26_ H_34_ O_12_	A	Hu et al., 2018
118	Hyuganoside IIIb	838845-08-2	C_26_ H_34_ O_12_	A	Hu et al., 2018
119	3,5-Dimethoxy-1-(3-hydroxypropen-1-yl)phenyl 4-O-α-L-rhamnopyranosyl-(1″→6′)-β-D-glucopyranoside	2171100-32-4	C_23_ H_34_ O_13_	A	Hu et al., 2018

A: fruits; B: leaves; C: stems; D: leaves and stems; E: peels; F: roots; G: leaves and fruits.

## Nutritional Composition

**Table 1** lists the nutritional composition of purple and yellow passion fruit juice reported from the USDA Food Composition Database. The data show evidence that the purple and yellow passion fruit juice contain a high percentage of carbohydrate, Vitamin A, Vitamin C, minerals, and fiber. In general, the nutritional composition content in purple passion fruit was basically the same as that in yellow variety. In general, the passion fruit has a potential to become a functional food.

## Pectin, Fiber, and Polysaccharides

Pectin, fiber, and polysaccharides are the most common functional ingredients in food products and exert a strong positive influence on human health. The contents of pectin, crude fiber and polysaccharides are 12.5%, 22.1%, and 20.62%, respectively (Wen et al., 2008). The seed of *P. edulis* is rich in insoluble dietary fiber (64.1%). After defatting, the insoluble fiber-rich fractions (84.9%–93.3%) including cellulose, pectic substances, and hemicellulose become the predominant components (Chau and Huang, 2003). GC-MS showed that polysaccharides from the peel of yellow passion fruit are composed of galacturonic acid (44.2%), arabinose (11.8%), glucose (11.8%), maltotriose (10.6%), mannose (9.0%), galactose (6.1%), xylose (3.6%), ribose (1.3%) and fucose (1.6%), and so forth. (Silva et al., 2012). Meanwhile, yellow passion fruit rind is a good source of naturally low-methoxyl pectin. Polysaccharide from purple passion fruit peel is mainly composed of galacturonic acid (80.32%), glucose (4.65%), ribose (4.41%), galactose (3.84%), arabinose (3.53%), mannose (1.34%), xylose (0.72%) and rhamnose (0.17%), and so forth. We found (1→4)-linked galacturonic acid is the main component of polysaccharides from yellow and purple passion fruit. Diverse studies have demonstrated that pectin and fibers from *P. edulis* peel can effectively eliminate free radicals such as DPPH and ABTS (Dos Reis et al., 2018), reduce cholesterol and blood glucose levels (Lacerda-Miranda et al., 2016), and obviously inhibit the growth of sarcoma180 (Silva et al., 2012), and so forth. Thus, the passion fruit peel may be utilized in the development of new fiber-rich healthy food products.

## Protein and Amino Acids

The total protein content from fruit pulp of passion fruit is 0.80 mg/g (Araujo et al., 2003). Importantly, some proteins in passion fruit have promising antifungal properties. For example, Pe-AFP1 (5.0 kDa), a 2S-albumin-protein-like peptide purified from the seeds of passion fruit, is found to be able to inhibit the growth of filamentous fungi *Trichoderma harzianum*, *Fusarium oxysporum*, and *Aspergillus fumigatus* with a respective IC_50_ values of 32, 34, and 40 μg/ml (Pelegrini et al., 2006). Free amino acids isolated from purple passion fruit mainly include leucine, valine, tyrosine, proline, threonine, glycine, aspartic acid, arginine, and lysine. Among them, lysine, threonine, leucine, and valine are indispensable amino acids for growth.

## Volatile Components

Volatile components should be the aromatic ingredients of passion fruit, and they also have anti-oxidative activity. The esters (59.24%), aldehydes (15.27%), ketones (11.70%), alcs. (6.56%), terpenes, and other miscellaneous compounds have been proven to exist in passion fruit (Narain et al., 2004). GC and GC-MS analysis revealed that the major volatile constituents of fruit shell of *P. edulis* Sims are 2-tridecanone (62.1%), (9Z)-octadecenoic acid (16.6%), 2-pentadecanone (6.2%), hexadecanoic acid (3.2%), 2-tridecanol (2.1%), octadecanoic acid (2.0%), and caryophyllene oxide (2.0%) (Arriaga et al., 1997). It is noteworthy that the volatile components changed during maturation.

## Lipids

*P. edulis* seeds contain 20% drying oil, solid fat acid 11.5% (palmitic and stearic acids) and 88.5% liquid acid (linolic and oleic acids). Seeds oil contain high amount of unsaturated fatty acids, and the major unsaturated fatty acids are linoleic acid (69.3%), oleic acid (14.4%), palmitic acid (10.1%), and stearic acid (2.9%) (Rana and Blazquez, 2008). The total content of crude oil in the residue of passion fruit after juice production is ∼24%.

## Minerals

Passion fruit is a very refreshing tropical fruit and full of minerals in fruit, juice, peel and seeds, which are known to be effective to human health. For instance, Fe, Zn, Mn, B, Cu, K, N, Ca, P, Mg, S, and Mo of skin and pulp and seeds of passion flower are 150, 41, 40, 25, 10, 3, 0.8, 0.4, 0.21, 0.15, 0.08, 0.08, and 110, 50, 16, 9, 6, 2, 1.4, 0.1, 0.25, 0.15, 0.08, and 0.12 ppm, respectively. We can consider passion fruit plant leaves as good resources of calcium and zinc due to the high content of both minerals. In the youngest leaves, the contents of N, P, K, and Zn are relatively high while the Ca, Mg, B, Cl, and Mn are relatively low (Freitas et al., 2007). **Table 1** shows passion fruit juice is a source of minerals that naturally rich in Ca, Mn, P, and K, and so forth. However, the information on harmful elements of passion fruit is rather scarce.

## Flavonoids

The passion fruit pulp is a famous food source of flavonoids, which contains 158.0 μg/ml of total flavonoids, 16.2 μg/ml of isoorientin (Zeraik and Yariwake, 2010) and 0.42 μg/g of quercetin (Rotta et al., 2019). The aerial parts of *P. edulis* extracted by reflux with 40% ethanol contain 0.90% of apigenin. So far, 33 flavonoids have been identified in various parts of *P. edulis* (Lutomski et al., 1975; Mareck et al., 1991; Moraes et al., 1997; Chang and Su, 1998; Xu et al., 2013). Among them, the major flavonoids identified from *P. edulis* are vitexin, isovitexin, isoorientin, apigenin, quercetine, luteolin, and their derivatives, which represent important classes of effective compounds in *P. edulis* regarding their various biological and pharmacological properties (Deng et al., 2010; Xu et al., 2013; Zhang et al., 2013).

## Triterpenoids

Twenty nine triterpenoids varying in chemical structures have been isolated from fruits, leaves, stems, and roots of *P. edulis* (Bombardelli et al., 1975; Yoshikawa et al., 2000a; Yoshikawa et al., 2000b; Zhou et al., 2009; Wang et al., 2013; Yuan et al., 2017). Cycloartane triterpenoids have showed the significant protective effects against damage of PC_12_ cell induced by glutamate, which can be used for the treatment of neurodegenerative disease (Xu et al., 2016). Cycloartane triterpenoids cyclopassiflosides IX and XI at 50 mg/kg displayed antidepressant-like effect (Wang et al., 2013).

## Alkaloids

Alkaloids including harmidine, harmine, harmane, harmol, *N*-*trans*-feruloyltyramine, and *cis*-*N*-feruloyltyramine have been found in fruits and leaves of *P. edulis* (Lutomski et al., 1975; Yuan et al., 2017). Harmine, a fluorescent harmala alkaloid, can reversibly inhibit monoamine oxidase A and angiogenesis and suppress tumor growth. Meanwhile, it showed anti-inflammatory activity by significantly inhibiting the NF-κB signaling pathway (Liu et al., 2017; Li et al., 2018).

## Sulforaphanes and Carotenoids

Six sulforaphanes and 13 carotenoids have been isolated and identified in fruits of *P. edulis* (Winter et al., 1976; Engel and Tressl, 1991; Mercadante et al., 1998). Carotenoids from vegetables and fruit play important roles in physiological functions, and thus have health benefits including anti-obesity, antidiabetic, and anticancer activities, and so forth. (Chuyen and Eun, 2017).

## Biological Activities

In China, South America, India, and so forth., *P. edulis* is commonly used as a tonic, digestive, sedative, diuretic, antidiarrheal, insecticide in traditional medicine for the treatment of cough, dry throat, constipation, insomnia, dysmenorrhea, colic infants, joint pain, and dysentery, and so forth. (Dhawan et al., 2004). Modern biochemical and pharmacological studies confirmed that the purified components and crude extracts from *P. edulis* showed a wide range of *in vitro* and *in vivo* bioactivities (Chau and Huang, 2005; Nayak and Panda, 2012; Kinoshita et al., 2013; Otify et al., 2015; Panchanathan and Rajendran, 2015; Silva et al., 2015; Dzotam et al., 2016; Zhang et al., 2016; Ayres et al., 2017; He et al., 2017; Goss et al., 2018; Liu et al., 2018; Mota et al., 2018; Panelli et al., 2018). **Table 4** shows the biological activities of main compounds isolated from *P. edulis*.

**Table 4:** Biological activities of compounds isolated from *P. edulis* ("↓", reduce; "↑", increase).

Bioactivity	Compound	Experiment	Biological results		References
			Positive control	Compound	
Anti-inflammatory effect	*α*-Tocopherylquinone	RAW 264.7 cells		IC_50_ = 34.92 μM, NO production↓	Hu et al., 2018
	Luteolin-8-*C*-*β*-digitoxopyranoside	RAW 264.7 cells		IC_50_ = 16.12 μM, NO production↓	Hu et al., 2018
	Luteolin-8-*C*-*β*-boivinopyranoside	RAW 264.7 cells		IC_50_ = 26.67 μM, NO production ↓	Hu et al., 2018
	Isoorientin	Swiss mice	Indomethacin (5 mg/kg), dexamethasone (0.5 mg/kg)	25 mg/kg ip., leukocytes, neutrophils, mononuclears↓, MPO activity↓	Zucolotto et al., 2009
	Vicenin-2	Swiss mice	Indomethacin (5 mg/kg), dexamethasone (0.5 mg/kg)	25 mg/kg ip., leukocytes, neutrophils, mononuclears↓, MPO activity↓	Zucolotto et al., 2009
	Spinosin	Swiss mice	Indomethacin (5 mg/kg), dexamethasone (0.5 mg/kg)	25 mg/kg ip., leukocytes, neutrophils, mononuclears↓, MPO activity↓	Zucolotto et al., 2009
	Orientin	DMH induced colorectal cancer in rats		10 mg/kg ip., TNF-α, IL-6, iNOS and COX-2 expression ↓	Thangaraj and Vaiyapuri, 2017
Neuroprotective effects	1α,3β-dihydroxy-16-keto-24(31)-en-cycloartane	PC_12_ cells		0.05-0.42 μM against the glutamate-induced neurotoxicity	Xu et al., 2016
	31-Methoxyl-passifloic acid	PC_12_ cells		0.06-0.23 μM against the glutamate-induced neurotoxicity	Xu et al., 2016
	Cyclopassifloside II	PC_12_ cells		0.08-0.35 μM against the glutamate-induced neurotoxicity	Xu et al., 2016
	Cyclopassifloside VIII	PC_12_ cells		0.06-0.46 μM against the glutamate-induced neurotoxicity	Xu et al., 2016
	Cyclopassifloside XIV	PC_12_ cells		0.08-0.32 μM against the glutamate-induced neurotoxicity	Xu et al., 2016
	Luteolin	PC12 cells		50.0 μM, NGF-induced neurite outgrowth ↑	Xu et al., 2013
	Piceatannol	mouse embryonic stem cells		2.5 µM, astrocyte differentiation↑	Arai et al., 2016
Anxiolytic-like effect	Isoorientin	Swiss albino mice	Diazepam (2 mg/kg Ig.)	40 and 80 mg/kg, time spent in open arms of the elevated plus-maze ↑	Deng et al., 2010
	Luteolin-7-O-[2-rhamnosylglucoside]	Swiss mice	Diazepam (1 mg/kg Ig.)	30 mg/kg, time spent in the open arms of the elevated plus maze test ↑	Coleta et al., 2006
Antidepressant-like effect	Cyclopassiflosides IX	ICR mice	Clomipramine (50 mg/kg)	50 mg/kg Ig., immobility time in forced swim and tail suspension test reduced by 22.72% and 39.26%	Wang et al., 2013
	Cyclopassiflosides XI	ICR mice	Clomipramine (50 mg/kg)	50 mg/kg Ig., immobility time in forced swim and tail suspension test reduced by 19.16% and 43.12%	Wang et al., 2013
Sedative-like activity	Isoorientin	Swiss albino mice	Diazepam (2 mg/kg Ig.)	40 mg/kg and 80 mg/kg, number of spontaneous activities↓	Deng et al., 2010
Vasorelaxation effect	Piceatannol	Isolated rat thoracic aorta		30 μM, eNOS expression↑	Sano et al., 2011; Kinoshita et al., 2013
	Piceatannol	Human EA. hy926 endothelial cells		20 μM, 48 h, eNOS expression↑	Kinoshita et al., 2013
	Scirpusin B	Isolated rat thoracic aorta		30 μM, endothelium-derived NO↑	Sano et al., 2011
Melanin inhibition and collagen synthesis promotion	Piceatannol	Dermal Cells (SF-TY cells)		4.5 μM, melanin synthesis↓; 5 μM increased collagen synthesis↑	Matsui et al., 2010
	Isoorientin	B16 melanoma cells		100 μM, melanin content (47.2% reduction) ↓	Zhang et al., 2013
	Chrysin 6-C-β-rutinoside	B16 melanoma cells		100 μM, melanin content (47.2% reduction) ↓, MITF, tyrosinase, TRP-1, and TRP-2 proteins levels ↓	Zhang et al., 2013
	(6S,9R)-roseoside	B16 melanoma cells		100 μM, melanin content (37.3% reduction) ↓	Zhang et al., 2013
Antidiabetic activity	Piceatannol	db/db mice		50 mg/kg, blood glucose levels↓	Uchida-Maruki et al., 2015
	Piceatannol	Humans		20 mg/day for 56 days, the insulin sensitivity, BP and HR improvement	Kitada et al., 2017
Antioxidant activity	Scirpusin B	DPPH	Trolox	5-40 μM, DPPH radical scavenging activities	Sano et al., 2011
	Piceatannol	DPPH	Trolox	5-40 μM, DPPH radical scavenging activities	Sano et al., 2011
	Piceatannol	BALB/cByJ Jcl mice		10 mg/kg Ig., 14 days, number of astrocytes↑	Arai et al., 2016

### Antioxidant Activity

Large amount studies highlight the potential of passion fruit as a valuable source of natural antioxidants which can eliminate free radicals or inhibit the activity of free radicals, thereby helping the body to maintain an adequate antioxidant status. The antioxidant and radical scavenging activities of the extracts from fruit, seed, peel, leaves, bark of *P. edulis* have been studied *via* ABTS (Zas and John, 2017), AAPH, DPPH, FRAP, ORAC, HOCI scavengers and ferrous ions assays *in vitro*, as well as several *in vivo* experiments (Rotta et al., 2019). The results showed that aqueous, ethanol, polyphenol-rich in particular extracts of leaf (Thomas et al., 2019), peel and seed from *P. edulis* demonstrate potential antioxidant and radical scavenging activities. *P. edulis* fruit showed a higher antioxidant activity (64% of DPPH reduced) than mango, pineapple, banana and litchi (45%–58%). In addition, passion fruit exerted a higher free radical-scavenging activity (14.08 μmol Trolox equivalent) than little banana, big banana, papaya Colombo, papaya solo, onion, nectarine, orange, mango american, pineapple, mango josé, and litchi (< 10 μmol Trolox equivalent). The variation of polyphenol components (286.6 mg gallic acid equivalent/100 g), total flavonoid (70.1 mg quercetin equivalent/100 g), carotenoid (3.8 mg β-carotene equivalent/100 g), vitamin C (44.4 mg ascorbic acid equivalent/100 g) may be responsible for the radical-scavenging activity (Septembre-Malaterre et al., 2016). Passion fruit seeds are rich in the total phenolic compounds, and show the highest antioxidant capacities in the FRAP assay (119.32 μmol FeSO_4_ g^-1^ DW) than pulp, raw peel, oven dried peel, lyophilized peel (27.16-60.27 μmol), while it showed antioxidant activity with the lowest IC_50_ value (DPPH) of 49.71 μmol than that of pulp (869.05 μmol), raw peel (347.56 μmol), oven dried peel (371.14 μmol), and lyophilized peel (225.29 μmol) (Morais et al., 2015). The *in vivo* treated the bark of *P. edulis* to obese male db/db mice could increase the antioxidant capacity of plasma, kidney, liver and adipose tissue, and reduce lipid oxidation of kidney and liver (Panelli et al., 2018). Furthermore, administration of *P. edulis* leaves, peel, and seeds to streptozotocin-induced diabetic rats showed antioxidant capacity by improving the anti-oxidants enzyme in animal visceral organs (da Silva et al., 2013; Kandandapani et al., 2015).

### Analgesic and Anti-Inflammatory Activities

#### Analgesic Activity

Comparative studies showed that n-butanol extracts of *P. edulis* leaves had a dose-dependent analgesic activity in a thermal stimulation pain model (Nayak and Panda, 2012). In acetic acid-induced writhing, formalin-induced paw licking and response latency in the hot plate test, the polysaccharide of the dried fruit of the *P. edulis* reduced acetic acid induced writhing and formalin-induced paw licking, but it did not produced a significant increase in reaction time in the hot plate test, suggesting that the analgesic activity of polysaccharide is related to peripheral mechanisms (Silva et al., 2015). However, detailed and accurate data on the possible molecular mechanism and bioactive compounds are need to be carried out.

#### Anti-Inflammatory Activity

Anti-inflammatory activity of *P. edulis* extracts has been evaluated through *in vivo* tests like the inflammation induced by 2,4,6-trinitrobenzenesulphonic acid, dextran sodium sulphate carrageenan (Herawaty and Surjanto, 2017), substance P, histamine, bradykinin, and dextran sodium sulphate (Cazarin et al., 2016), and so forth. In a 2,4,6-trinitrobenzenesulphonic acid induced rat colitis model, the aqueous extract of *P. edulis* leaves reduced pro-inflammatory levels of IL-1β and TNF-α (Cazarin et al., 2015). In a dextran sodium sulphate caused mice colitis model, *P. edulis* peel flour reduced pro-inflammatory cytokine TNF-α, IL-1β, IL-6, IL-12, and IL-17 expression and decreased the expression of MCP-1 and ICAM-1 (Cazarin et al., 2016). This could be attributed to the presence of bioactive compounds like C-glycosyl flavonoids vicenin, orientin, isoorientin, vitexin and isovitexin. Intraperitoneal injection of the polysaccharide from the dried fruit of *P. edulis* at the dose of 3 mg/kg reduced mice paw oedema induced by the compound 48/80, carrageenan, histamine, serotonin, and prostaglandin E_2_, and significantly reduced vascular permeability, TNF-α and IL-1β level (Silva et al., 2015).

### Antimicrobial Activity

The passion fruit possesses antifungal and antibacterial activity against fungi and bacteria which cause infectious diseases in human and plants. The peptide with close similarity to 2S albumins from passion fruit seeds has antifungal properties against *Trichoderma harzianum*, *Fusarium oxysporum*, *Aspergillus fumigatus*, *Colletotrichum lindemuthianum*, *Kluyveromyces marxiannus*, *Candida albicans, Candida parapsilosis* and *Saccharomyces cerevisiae* (Agizzio et al., 2003; Pelegrini et al., 2006; Ribeiro et al., 2012; Jagessar et al., 2017). The methanol extracts of pericarp of *P. edulis* inhibited the growth of different bacterial strains such as *Escherichia coli*, *Enterobacter aerogenes*, *Klebsiella pneumoniae*, *Pseudomonas aeruginosa*, and *Providencia stuartii* with minimum inhibitory concentrations ranging from 128 to 1024 μg/ml. This could be due to the presence of bioactive compounds such as polyphenols triterpenes, and sterols contained in the methanol extracts (Dzotam et al., 2016). Indeed, in a systematic study, Ramaiya et al. (2014) showed that the total phenolic and antioxidant contents had significantly antibacterial properties. Oil from yellow passion fruit seeds showed antibacterial activity against *Escherichia coli, Salmonella enteritidis, Staphylococcus aureus* and *Bacillus cereus. n*-hexane, tocopherol, linoleic acid, unsaturated fatty acids were identified as major compounds in the oil (Pereira et al., 2019). However, *in vivo* and clinical studies are needed for confirmation.

### Anti-Hypertensive Activity

The anti-hypertensive activity of both yellow and purple passion fruit products has been proved in spontaneously hypertensive rats. Oral administration of *P. edulis* peel extract reduced hemodynamic parameters, decreased serum nitric oxide level (Zibadia et al., 2007), and lowered blood pressure in spontaneously hypertensive rats (Ichimura et al., 2006; Lewis et al., 2013). This could be attributed to the polyphenols such as luteolin, luteolin-6-C-glucoside, quercetin, edulilic acid, ascorbic acid, piceatannol (Kinoshita et al., 2013) and anthocyanin, and so forth. which can mediate nitric oxide modulation and have potent vascular effects (Ichimura et al., 2006; Lewis et al., 2013; Konta et al., 2014). However, the exact mechanisms and compounds responsible for this effect need further investigation.

### Hepatoprotective and Lung-Protective Activities

Oral administration of purple passion fruit peel extract showed hepatoprotection against chloroform (1 mmol)-induced rat liver injury (Zibadia et al., 2007), and showed noteworthy hepatoprotective activity against CCl_4_ induced hepatotoxicity (Kavitha et al., 2016). In ethanol-induced liver injury, treated daily with fruit juices to mice for 15 days could protect ethanol-induced liver injury by decreasing AST and ALT in liver, and alleviating the inflammation, oxidative stress (Zhang et al., 2016). In addition, the passion fruit seed extract prevented non-alcoholic fatty liver disease by improving the liver hypertrophy and hepatic histology of the high-fat diet-fed rats (Ishihata et al., 2016). In a pulmonary fibrosis of C57BL/6J mice model induced by bleomycin, administration of passion fruit peel extract significantly reduced loss of body weight and mortality rate, decreased the count of inflammatory cells, macrophages, lymphocytes, and neutrophils, reduced MPO activity and restored bleomycin induced depletion of SOD activity.

### Hypolipidemic Activity

Hyperlipidemia can directly cause some diseases that seriously endanger human health, such as atherosclerosis, coronary heart disease, pancreatitis, and so forth. Passion fruit plays an important role in preventing hyperlipidemia. The passion fruit juice at a dose of 580 mg/kg once a day for 30 consecutive days significantly reduced total cholesterol, triglyceride, and low-density lipoprotein cholesterol levels, and increased high-density lipoprotein cholesterol level in diabetic Wistar rat offspring (Barbalho et al., 2011), and peel flour of *P. edulis* counteracted cumulative body weight gain, decreased adiposity and leptin level, increased adiponectin in diet-induced obesity in rat (Lima et al., 2016). Oral administration of pectin from *P. edulis* fruit peel 0.5–25 mg/kg for 5 days effectively decreased triglyceride levels in diabetic rats (Silva et al., 2011), and the insoluble fiber derived from seed of *P. edulis* decreased serum triglyceride and total cholesterol, liver cholesterol, and increased the cholesterol, total lipids, and bile acids levels in feces of golden Syrian hamsters (Chau and Huang, 2005).

### Antidiabetic Activity

Diverse studies have demonstrated that peel flour, juice, and seeds of *P. edulis* showed antidiabetic potential effects by reducing glucose tolerance in diabetic mice, and rats. Oral administration of passion fruit juice at a dose of 580 mg/kg once a day for 30 consecutive days significantly reduced glucose in streptozotocin induced diabetic rat offspring (Barbalho et al., 2011), and administration of passion fruit seed or leaf extract also reduced the blood glucose levels of db/db mice, alloxan induced diabetes mellitus in Wistar albino rats or streptozotocin (STZ) induced diabetic rats (Kanakasabapathi and Gopalakrishnan, 2015; Panchanathan and Rajendran, 2015; Uchida-Maruki et al., 2015). Oral administration of pectin from *P. edulis* fruit peel at a dose of 0.5–25 mg/kg daily for 5 days lowered blood glucose in diabetic rats induced by alloxan, providing a new treatment for type 2 diabetes (Silva et al., 2011). Peel flour of *P. edulis* intake increased glucose-dependent insulinotropic polypeptide and glucagon-like peptide-1, improved the insulin sensitivity in high-fat diet-induced obesity rats by increasing the glucose disappearance rate (Lima et al., 2016), and also prevents insulin resistance induced by low-fructose-diet in rats. In addition, the leaf extract of *P. edulis* full of flavonoids also has a health benefit to the diabetic state, and show the prevent effect on the appearance of its complications (Salles et al., 2020; Soares et al., 2020).

### Antidepressant Activity

Antidepressant potential of stems and leaves extracts has been certified *in vivo*. Oral administration of ethanol extracts of the aerial parts (equal to 10 and 2 g/kg of the plant materials) of *P. edulis* to mice for 7 day exhibited antidepressant-like effect *via* reduced immobility time in the forced swim and tail suspension tests in mice. Further evidence showed that the cycloartane triterpenoid cyclopassiflosides IX and XI at the dosage of 50 mg/kg possessed an antidepressant-like effect, which indicated that those cycloartane triterpenoids may be the main responsible bioactive compounds of *P. edulis* (Wang et al., 2013). Oral administration of the aqueous (300 mg/kg), ethyl acetate (50 mg/kg), and butanol extracts (50 mg/kg) of *P. edulis* Sims fo *edulis* reduced the immobility time in the mice forced swimming test, which is similar to nortriptyline and fluoxetine. Particularly, ethyl acetate and butanol extract rich in flavonoids showed preferably the antidepressant effects, and that can be counteracted by p-chlorophenylalanine, α-methyl-DL-tyrosine chloride and sulpiride, suggesting this action is related exclusively to regulate serotonergic and dopaminergic transmission such as 5-HT, catecholamine and D_2_ receptor (Ayres et al., 2017).

### Anxiolytic-Like Activity

The fragrant fruits and their twigs and leaf of *P. edulis* Sims are most used as a folk medicine in treating anxiety in American countries. *In vivo* data suggest that varieties of crude extracts like butanolic, methanol, ethanol, hydro-ethanol, and aqueous extract showed anxiolytic-like effect in the model tested. The aqueous extract of *P. edulis* at 50, 100, and 150 mg/kg showed anxiolytic-like effects in the elevated plus-maze and inhibitory avoidance tests in rat. More importantly, administration of the aqueous extract of *P. edulis* did not disrupted rat memory process in an habituation to an open-field test, but diazepam impaired rat habituation with a simple modification of the open-field apparatus (Barbosa et al., 2008).

The methanol extract of aerial parts of *P. edulis* Sims at an oral dose of 75 mg/kg showed anxiolytic activity on the elevated plus-maze model of anxiety in mice, but oral dose of 125 mg/kg did not evoke any significant activity. Whereas, oral at higher doses of 200 and 300 mg/kg showed a mild sedative effect (Dhawan et al., 2001). Pre-treatment with 50, 100, and 150 mg/kg hydroethanol extracts and 400 and 800 mg/kg of spray-dried powders of *P. edulis* leaves also showed anxiolytic activity in the elevated plus-maze test in mice. It was suggested that the therapeutic effect of these extracts was due to the presence of a wide range of flavonoids such as isoorientin, orientin, luteolin, apigenin, and chrysin or their glycosides, and so forth (Petry et al., 2001; Coleta et al., 2006; Otify et al., 2015).

### Sedative Activity

There is cumulative evidence to suggest that *P. edulis* possess sedative activity, which are certified its therapeutic applications in insomnia in traditional folk medicines. Oral administration of aqueous extracts of pericarp and the leaves (300 mg/kg, 600 mg/kg and 1,200 mg/kg) of *P. edulis* f. flavicarpa Degener showed a rapid onset of action and a significant decrease dose-dependently in locomotor activity in C57BL/6J mice using radiotelemetry. It is noteworthy to mention that aqueous extracts of pericarp showed more significant effects on locomotor activity while compared to leaves extracts (Klein et al., 2014). 300 mg/kg n-BuOH and ethanolic extract of aerial part of *P. edulis* f. flavicarpa Degener hindered motor activity of mice, showing a sedative-like effect. Flavonoids, especially, isoorientin were identified as a major sedative constituent in the extract (Deng et al., 2010). The aqueous extracts mainly contain C-glycosylflavonoids isoorientin, vicenin-2, spinosin, and 6,8-di-C-glycosylchrysin. In addition, ethanolic extract is composed of flavonoids like isovetexin, apigenin-6-C-β-D-glucopyrano-4′-O-α-L-rhamnopyranoside, luteolin 6-C-β-D-chinovoside, and luteolin 6-C-β-D-fucoside (Zhou et al., 2009). Thus, the flavonoids may be responsible for the sedative activity of *P. edulis* f. flavicarpa Degener.

### Antitumor Activity

Most of the pharmacological work has been carried out on the antitumor activity of *P. edulis. In vitro*, the different varieties of extracts of *P. edulis* showed cytotoxicity against HepG_2_ (Aguillón et al., 2018), MCF-7, SW480, SW620, Caco-2 (Ramirez et al., 2017; Sandra et al., 2017; Mota et al., 2018), CCRF-CEM, CEM/ADR5000, and HCT116 [p53(-/-)] (Kuete et al., 2016). It was found that higher content of polyphenolic and polysaccharide contained in ethanolic extract may be related to the inhibition of matrix-metalloprotease MMP-2 and MMP-9 (Puricelli et al., 2003). *In vivo*, the ethanol extract of yellow passion fruit inhibited tumor growth with an inhibition rate of 48.5% and increased mice lifespan to nearly 42% in male Balb/c mice inoculated with Ehrlich carcinoma cells. This could be attributed to the presence of medium and long chain fatty acids such as lauric acid (Mota et al., 2018). Oral or intraperitoneal administration of the polysaccharide showed the inhibition of the growth of sarcoma 180 tumors with an inhibition ratio ranging from 40.59% to 48.73% (Silva et al., 2012).

## Clinical Effectiveness in Humans

Although the use of *P. edulis* has a key role in management of various ailments in folk medicine and in various preclinical experiments, the efficacy of this plants has not been explored in depth in human clinical trials. So far, few clinical trials of *P. edulis* have been conducted to determine if improved chronic diseases such as diabetes, hypertension, and asthma. An open, prospective, randomized clinical trial studies showed that combined use of the yellow passion fruit peel flour and hypoglycemic drugs like glyburide, metformin, and insulin, and so forth. exerted a favorable effect on insulin sensitivity during the 60 days period in type 2 diabetes mellitus patients (de Queiroz Mdo et al., 2012), but the single use of flour made from the rind of the yellow passion fruit over 56 days did not significantly improve glycemic control on type 2 diabetes patients (de Araújo et al., 2017). This seems to be inconsistent with the results of animal experiments, so it remains to verify the antiglycation effect using a multitude of reliable experimental probes and to explicit which type of chemical composition is mostly responsible for promoting the activity of hypoglycemic agents. In 28 days randomized, placebo-controlled, double-blind clinical trial, orally administered of purple passion fruit peel extract at 400 mg/day significantly decreased systolic and diastolic blood pressure by 30.9 and 24.6 mmHg, respectively (Zibadia et al., 2007). Oral administration of purple passion fruit peel 150 mg/day in 28 days clinical trial can effectively alleviate the clinical symptoms of cough by reducing wheeze and cough and improving shortness of breath in adults with asthma, and no adverse effects were found in this study (Watson et al., 2008). Meanwhile, oral administration of purple passion fruit peel 150 mg/day for 56 days in clinical trial substantially alleviated osteoarthritis symptoms, and its beneficial effects may be due to the anti-inflammatory properties (Farid et al., 2010). In general, these interesting studies may contribute to a better understanding of clinical efficacy of *P. edulis*. However, in consideration of the significant in-vitro and in vivo pharmacological activities of *P. edulis*, more randomized, double‐blind, placebo‐controlled and cross‐over studies are urgently needed.

## Toxicity

Many researches show that passion fruit does not cause any harmful side effects. *In vivo* acute and subacute toxicity studies indicate that oral administration of the ethanol extract of unripen fruit peel of *P. edulis* at the dose of 550 mg/kg had no toxic effect on the rats. Administration of the aqueous extract of *P. edulis* leaf was found to be safe even at the dose of 2,000 mg/kg. Importantly, mice behavioral pattern and hematologic parameters including RBC, Hb, WBC, MCV, MCH, platelets, neutrophils and lymphocytes had no abnormal change (Anurangi and Shamina, 2018). The subacute study showed that the aqueous extract was safe on the bone marrow function and it was neither hepatotoxic nor nephrotoxic (Devaki et al., 2012). These results provide a basis to further explore the clinical uses of passion fruit. However, more and extensively studies are still needed on its bioavailability and toxicity in animals and humans.

## Processing and Applications

Usually, passion fruit with an intense aroma can be eaten directly. Passion fruit is liable to deteriorate and has a short shelf life. Packaging with high oxygen (90%) atmosphere can effectively inhibit respiration and peel shape, keep vitamin C and solubility solids content, and increase total phenols content in passion fruit, improving the postharvest quality of passion fruit (Chen et al., 2018). Different processing methods affected the composition and activity of passion fruit. Steaming and boiling compared to frying protect the health-promoting properties of passion fruit (Gunathilake et al., 2018b). Today, passion fruit has been processed into a range of products on the basis of the attractive health effects and full of phytonutrients. A wide range of products made with passion fruit has been developed including cake, ice cream, jam, jelly, yoghurt, compound beverage, tea, wine, vinegar, soup-stock, condiment sauce, and so on. Additionally, many kinds of passion fruit drink with a refreshing taste are being prepared combining with Chinese medicines including *Lycium barbarum* and *Dendranthema morifolium*, and so forth, and are suitable for people to drink in the light of the vitamin supplementation, strengthening immunity, nourishing skin, and resisting fatigue (Zhang, 2018). Seed oil of *P. edulis* may be developed as functional food such as tea cream with whitening and anti-wrinkle effects. The nutritional ingredients and biological properties of *P. edulis* provide a strong basis for the development of *P. edulis* based food products.

The pulp of passion fruit can be both eaten or commonly juiced. The juice is often added to other juices to enhance its aroma. The passion fruit peel accounts for about 51% the fruit wet weight. Because of the large amount of fruit juice production, many thousand tons of seeds, pulps, and peels as agricultural coproducts during juice extraction. Peels, as the major wastes, have become an important burden on the environment. With the development of economy and people’s awareness of environmental protection, peel has been used extensively in the industrial production of pectin. It is a widely used functional food raw material with high value in reducing cholesterol levels, reducing hyperlipidemia, and hypertension, improving glucose tolerance and insulin response, helpful to gastrointestinal health and the prevention of some cancers. Pectin is used as a nutritional fiber delivery, gelling agent, and edible coating and stabilizer in the pharmaceutical, cosmetic and food industry, especially in the production of confectionery, jelly, and other products (de Souza et al., 2018). Pectin-based edible coating plays a waterproof role in fruit preservation, which have the functions of preventing moisture transfer and flavor loss, and improving hardness. The production of commercial pectin from passion fruit peel, seeds and bagasse could not only eliminate the problem of waste, but also provide a new source for pectin industry.

## Conclusions and Future Research Directions

Passion fruit is most popular for its attractive nutritional and sensory qualities to the health and well beings of the worldwide consumer. Secondary metabolites in passion fruit have been attracting considerable attention because these compounds exert numerous health benefits and economic value, and thus have been used in nutrition, cosmetics and medicine. Passion fruit and its by-products full of various chemical constituents and phytonutrients including polyphenols, dietary fiber, pectin, carotenes, and vitamin. The chemical constituents and properties of different kinds of passion fruit are diverse. Yellow passion fruit presented higher water and relatively lower nutritional components, while purple fruit presented higher content of vitamin C, vitamin A, fiber and calcium. Different plant parts (leaves, buds, peels, and pulp) and growth stages of *P. edulis* contain a variety of bioactive components such as total dietary and polyphenols. The yellow passion fruit presented higher content of pectin in peels, high content of carotene, quercetin, and kaempferol in pulps and higher values of total dietary fiber in seeds. The purple fruit was highlighted by a great value of anthocyanins in peels and seeds. Passion fruit peels as food waste account for 50% of the total fruit has also a high potential to obtain functional ingredients because it rich in biological active ingredients. Because of the unique bioactive constituents in passion fruit, diverse nutritional and medical benefits have been watched and recorded. The various extracts from different parts of *P. edulis* exhibited numerous pharmacological activities including antioxidant, analgesic and anti-inflammatory, antimicrobial, anti-hypertensive, hepatoprotective and lung-protective, anti-tumor, antidiabetic, hypolipidemic, antidepressant and anxiolytic-like capacities, and thus are used in phytotherapeutic remedies. In particular, acute toxicity and subacute toxicity studies have shown that a rationalized daily dose of passion fruit is probably safe for consumption. These outstanding results suggest that passion fruit may offer a range of health benefits, such as managing inflammatory and neurological disease, and also preventing some chronic diseases like hypertension and hyperlipidemia.

There are also research opportunities to better utilize passion fruit and its by-products for human consumption. Both passion fruit and its by-products are a rich source of polyphenols, so it is very important to optimization of appropriate processing methods to stabilize and improve the quality of these processed product. The structure and characteristics of polysaccharides in fruits remains to be studied. Pesticides may be present on the fruit and should be strictly monitored and controlled. Influence of genetic diversity, processing approach and living environment on chemical composition and nutritional value of passion fruit should be further explored. Studies on varieties of *P. edulis* are very limited, especially the confusion between *P. edulis* and *P. incarnata* remains, that need to be attracted special attention from botanist. The pharmacological activity reports of the *P. edulis* plant are mostly based on preliminary evaluation, and the models used lack appropriate standards or reasonable dosage. *P. edulis* has showed therapeutic potentials as *in vitro* anticancer agent against various tumor cell lines, but *in vitro* cytotoxic activities need to be supported with the *in vivo* studies and clinical trials to confirm its role as an anticancer agent in future. The structure-activity relationship and molecular mechanism of bioactive compounds or crude extracts of *P. edulis* will also be the focus of future research and practice. More importantly, clinical trial on *P. edulis* efficacy and safety are very scarce to support claims of efficacy.

## Author Contributions

YY, MW, MZ and JF obtained the literatures. FL, ZZ, and XH wrote the manuscript. XH, YL, and ZW gave ideas and edited the manuscript. All authors approved the paper for publication.

## Conflict of Interest

The authors declare that the research was conducted in the absence of any commercial or financial relationships that could be construed as a potential conflict of interest.

## Abbreviations

5-HT, 5-hydroxytryptamine; AAPH, 2,20-azobis[2-methyl-propionamidin] dihydrochloride; ABTS, 2′-azino-bis (3-ethylbenzthiazoline-6-sulfonic acid); ALT, alanine aminotransferase; AST, aspartate aminotransferase; BP, blood pressure; Caco-2, human cloned colon adenocarcinoma cells Caco-2; CCl_4_, carbon tetrachloride;COX-2, cyclooxygenase-2; DMH, 1,2-di-Me hydrazine; DPPH, 2,2-diphenyl-1-picrylhydrazyl; eNOS, endothelial nitric oxide synthase; FRAP, ferric ion reducing antioxidant power; GC-MS, gas chromatography-mass spectrometer; GC-O, gas chromatography-olfactometry; Hb, hemoglobin; HOCI, hypochlorite; HPLC, high performance liquid chromatography; HR, heart rate; HRGC-MS, high resolution gas chromatography-mass spectrometry; ICAM-1, intercellular cell adhesion molecule-1; IL-12, interleukin-12; IL-17, interleukin-17; IL-1β, interleukin-1β; IL-6, interleukin-6; iNOS, inducible nitric oxide synthase; Ki67, antigen KI-67; LC-DAD-ESI-MS, liquid chromatography-diode array detector-electrospray ionization/mass spectrometric; MCF-7, human breast adenocarcinoma cell line; MCH, mean corpuscular hemoglobin; MCP-1, human monocyte chemoattractant protein-1; MCV, erythrocyte mean corpuscular volume; MMP-2, matrix metallopeptidase 2; MMP-9, matrix metallopeptidase 9; MPO, myeloperoxidase; NO, nitric oxide; NGF, nerve growth factor; ORAC, oxygen radical absorbance capacity; PCNA, proliferating cell nuclear antigen; RBC, red blood cell; SOD, superoxide dismutase; STZ, streptozotocin; SW480, human colon cancer cell line SW480; TNF-α, tumor necrosis factor-α; UHPLC-MS/MS, ultraperformance liquid chromatography-tandem mass spectrometry; WBC, white blood cell.
